# Microgravity’s effects on miRNA-mRNA regulatory networks in a mouse model of segmental bone defects

**DOI:** 10.1371/journal.pone.0313768

**Published:** 2024-12-02

**Authors:** Aarti Gautam, Nabarun Chakraborty, George Dimitrov, Allison Hoke, Stacy Ann Miller, Kevin Swift, Bintu Sowe, Carolynn Conley, Melissa A. Kacena, Rasha Hammamieh

**Affiliations:** 1 Medical Readiness Systems Biology, Center for Military Psychiatry and Neuroscience, Walter Reed Army Institute of Research, Silver Spring, Maryland, United States of America; 2 General Dynamics Information Technology, Vysnova Partners, Landover, Maryland, United States of America; 3 Oak Ridge Institute for Science and Education, Oak Ridge, Tennessee, United States of America; 4 Space Test Program, Houston, Texas, United States of America; 5 Department of Orthopaedic Surgery, Indiana University School of Medicine, Indianapolis, Indiana, United States of America; 6 Richard L. Roudebush VA Medical Center, Indianapolis, Indiana, United States of America; Lorestan University, ISLAMIC REPUBLIC OF IRAN

## Abstract

Rehabilitation from musculoskeletal injuries (MSKI) complicate healing dynamics typically by sustained disuse of bone and muscles. Microgravity naturally allows limb disuse and thus an effective model to understand MSKI. The current study examined epigenetic changes in a segmental bone defect (SBD) mouse model in a prolonged unloading condition after spaceflight (FLT). We further connected potential miRNA–mRNA regulatory pathways impacting bone healing. Here, SBD surgery was performed on nine-week-old male mice that were launched into space for approximately 4 weeks. Sham with no surgery and ground controls were included in the study. The midshaft of the ipsilateral femur (with callus on the surgical mice) as well as the ipsilateral quadriceps tissue were used for analysis. Femur and quadriceps had a distinct miRNA profile. There was a stronger surgery effect as observed by miRNA expression when compared to microgravity effects. Leukopoiesis, granulopoiesis, myelopoiesis of leukocytes, differentiation of myeloid leukocytes, and differentiation of progenitor cells were all altered because of surgery in the femur. The biological functions such as apoptosis, necrosis, and activation of cell migration and viability were altered because of surgery in quadriceps. Integrating the transcriptome and microRNA data indicated pronounced changes because of microgravity. According to pathway analysis, microgravity had a greater impact on the quadriceps tissue than the bone tissue in the absence of surgery. The altered biological functions resulting from microgravity were validated by integrating limited proteomics data to miRNA-mRNA. Thus, this study highlights the importance of dynamic interplay of gene-epigene regulations as they appear to be intrinsically interconnected and influence in combination for the biological outcome.

## Introduction

Musculoskeletal injury (MSKI) is any injury that affects the bones, muscles, ligaments, nerves, or tendons resulting in pain. Approximately 1.71 billion people globally live with musculoskeletal conditions including: fractures, osteoarthritis, amputation, and rheumatoid arthritis [1]. Moreover, MSKI account for 61 million people treated in health-care centers in the United States alone [2]. MSKIs often have long healing periods that are commonly associated with complications which can have serious and long-lasting effects (e.g., chronic disability and dysfunction that result in direct and indirect physical and financial burdens). Segmental bone defects (SBD) are conditions where segment of the bone is missing or damaged due to trauma, infection or surgical removal and cannot be expected to heal on their own compared to a narrow gap resulting from a fracture which can be set by a cast [3]. Modern techniques have made headway allowing severely injured limbs with SBD to be saved. However, maintaining satisfactory function and/or delayed healing is still a challenge and 5–10% of fractures result in non-unions. The underlying mechanisms which prevent SBD from naturally healing are not well understood and it is necessary to identify novel approaches for clinical interventions that promote bone healing process.

Bone healing involves several overlapping phases such as inflammation, repair and finally, bone remodeling [4] and is governed by intricate molecular processes [5, 6]. Genes associated with bone healing encodes for various proteins involved in cell-proliferation, differentiation, and extra cellular matrix synthesis. Additionally, increasing evidence shows that noncoding RNAs are important regulators of chondrogenesis, osteogenesis and fracture healing [7–9]. Using simulated microgravity conditions, many miRNA were identified to be linked between microgravity-induced skeletal muscle atrophy and immune function deregulation [10].

Preclinical models such as mice, rats, rabbits, sheep, and equine have been used mimicking human injuries [11, 12] and their immediate weight bearing tendency post SBD confounds its outcomes. Microgravity, as observed in space is an unique environment with absence of gravitational loading on bones and muscles offers a rare setup to study the unloaded musculoskeletal system [13]. Some simulation models mimicking microgravity-like conditions do exist however there are potential differences between simulated microgravity and real space environment [14]. Conducting mice studies at International Space Station (ISS) is now possible because of rodent research facility setup where even live animal return is now possible [15–17] as snap freezing euthanized mice was the only solution for previous studies [18–27]. Sample preservation and collection timing to maintain transcriptional and other molecular information in resource-constrained environment such as those found in space [28] have been conducted by multiple labs. Multi-omic studies [18–20] have been conducted on the sample retrieved from frozen carcasses have demonstrates that tissues collected post-thawing can be a good option to study molecular mechanisms such as miRNA [29, 30] mRNA [31–33]. Metabolites [31, 32] and proteins [34, 35].

In the current study, we examined miRNA expression in bone and muscle tissue in response to microgravity, SBD surgery, and its combination. These samples were collected from the RR-4 (Rodent Research 4) mission that was launched on February 19, 2017, on SpaceX CRS-10 (Commercial Resupply Services) [31, 32]. In this mission, mice were launched into space four days post SBD surgery [36, 37]. Previously, musculoskeletal tissues such as quadriceps muscle have been tested using different preservation methods where gene expression patterns were found to be less sensitive to preservation methods [28]. Also, exposure to microgravity has been shown to decrease bone mineral density is known to be affected because of host microbiome and metabolites [17]. We hypothesized that the self-healing process of SBD in a prolonged unloading condition differentially perturbs not only the bone genomic and epigenomic landscape but also connected muscle tissue. We explored pathways and networks perturbed by miRNA-mRNA integrative analysis as the mRNA data have been previously published [31, 32]. Selected pathways and networks affected such as apoptosis and cell proliferation with clinical translational potential. The data herein provide emphasis on gene-epigene relationship and provide insights into the underlying mechanisms of bone healing that may help to explore new biological pathways to optimize future intervention strategies.

## Materials and methods

### Animals

All experiments were performed in accordance with the National Institute of Health Guide for the Care and Use of Laboratory Animals and followed experimental protocols approved by NASA Animal Care and Use Committees, and Animal Care and Use Review Office of U.S. Army Medical Research and Development Command. All mice related procedures were carried out as reported previously [31, 32, 37–39]. Seven-week-old male C57BL/6J mice were purchased from Jackson Laboratories (Bar Harbor, ME, USA) and were received two weeks prior to their launch. The mice were acclimated to the new caging/environment including the special lixit water bottles and Nutrient-upgraded rodent food bars for approximately 10 days, and were ear punched for identification.

### Mice groups

The mice were randomly assigned four groups (n = 10/group): 1) Sham surgery on Ground (GRD-Sham); 2) Sham surgery housed in FLT (FLT-Sham); 3) SBD surgery on Ground (GRD-Surgery); and 4) SBD surgery housed in the FLT (FLT-Surgery). Surgical procedures were carried out four days before launch as previously described [36, 38, 39]. Flight mice were housed in rodent habitat at ISS [24, 25].

### Segmental bone defect surgery

Mice were anesthetized using Ketamine-Xylazine (12.5–20 mg/kg) followed by a one cm incision was then made over the right femoral midshaft extending to the knee joint. The knee was flexed, and a 27-gauge hypodermic needle was inserted into the distal end of the femur and passed in a retrograde fashion into the intramedullary canal. A 2mm segment from the midpoint of the femoral diaphysis was removed and a 2 mm poly (propylene fumarate)/tricalcium phosphate graft was inserted to maintain the defect size. The needle tip was used to stabilize and align the femoral defect followed by cutting the distal end of the femur. A saline-soaked collagen sponge (RCM6 Resorbable Collagen Membrane, ACE, Brockton, MA, USA) was wrapped around the scaffold and sutured into place. The muscle was closed with simple interrupted Vicryl suture, and the skin was closed using wound clips (7mm, Braintree Scientific, Braintree, MA, USA). The mice were provided with buprenorphine (0.05mg/kg) for analgesia once they recovered from anesthesia and then were returned to their original cages with K3392 Rest Stops (Bio-Serve, Flemington, NJ, USA) for the first 2 days to assist with recovery. Two days prior to launch, the FLT-Sham and FLT-Surgery mice were transferred into the Transporter of the SpaceX Dragon capsule followed by launch on. February 19, 2017. Ground control timeline duration was identical but was shifted to the right 5 days. Mice were euthanized between L + 24 and L + 28 days. Euthanasia was performed by injection of ketamine/xylazine (150/45 mg/kg) followed by closed chest cardiac puncture with blood withdrawal and cervical dislocation.

### Tissue sample collection

**Fig 1:**
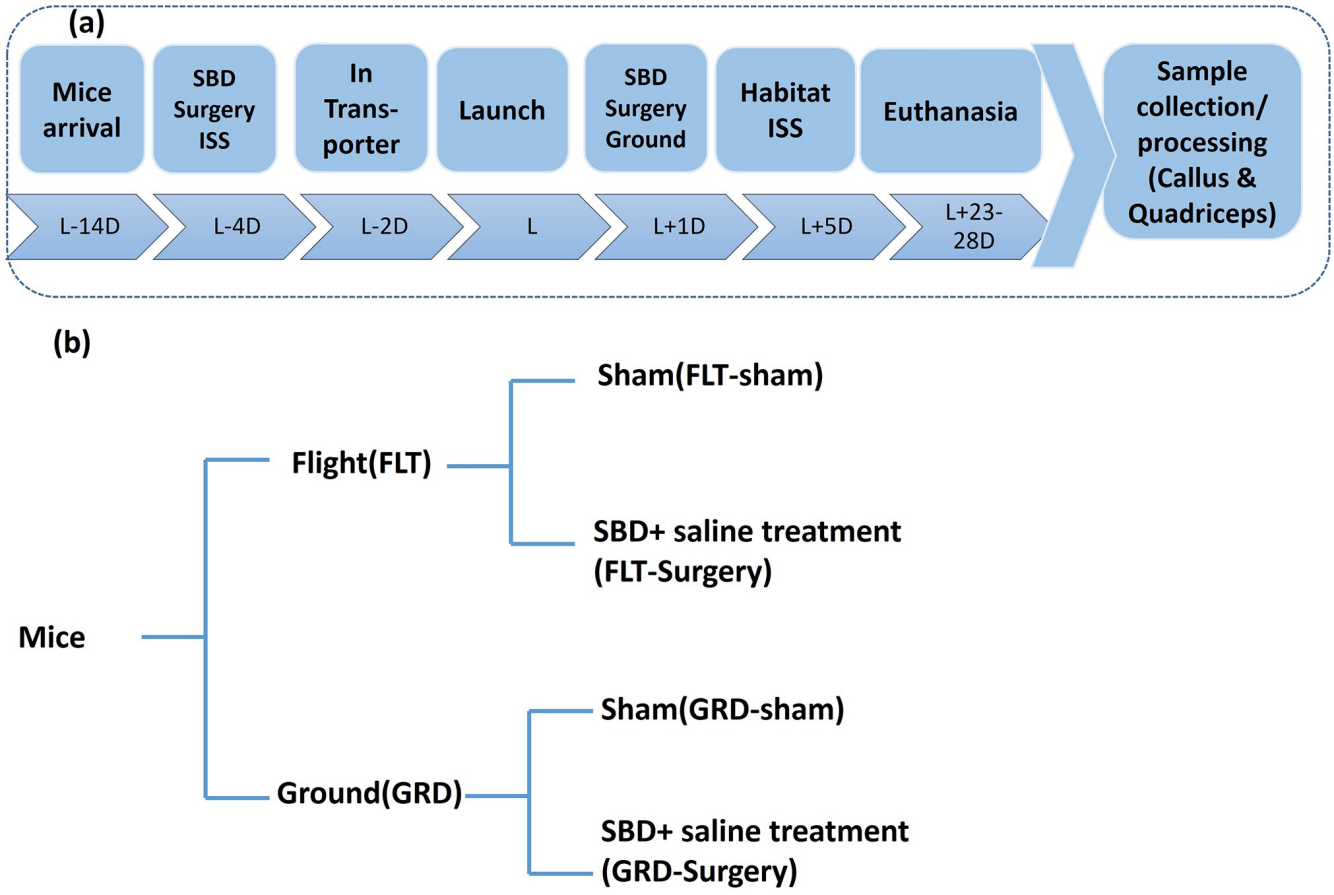
Timeline of the study. (a) Seven-week-old C57BL/6J mice arrived in facility two weeks before the launch into International Space Station (ISS). Four days before the launch they were operated for segmental bone defect (SBD) surgery on the mice that were launched into ISS. Four weeks post-surgery, the mice were euthanized to preserve the carcass at their respective timepoints. (b) With the two groups 1) Flight and 2) Ground; two subgroups were generated; one subgroup underwent surgery with saline treatment and other served as unoperated shams. The callus and quadriceps tissues were collected from four groups of mice 1) FLT-Sham; 2) FLT-Surgery; 3) GRD-Sham; and 4) GRD-Surgery. Each group had sample size of n = 5 mice. (L: Launch).

The mice were euthanized approximately 28 days post-launch at approximately 13 weeks of age and were stored in aluminum foil at or below -80°C until they arrived at the laboratory on Earth approximately 2 weeks after euthanasia. Partially thawed carcasses were used for tissue collection. For the current study, the proximal and distal 2mm of the femur of surgery mice (both FLT and GRD) were removed that included bone and bone marrow and the remainder of the midshaft was considered the callus regions. The uninjured quadriceps tissue was also collected for the current study. The same regions of sham animals (both FLT and GRD) were saved as controls. All dissections were performed on the same day in the laboratory setting and there were maximum of 5 mice/group (except n = 4 for FLT-SAL group as one animal had to be euthanized earlier) allotted for multi-omics assays. The timeline of this study is shown as Fig 1.

### RNA extraction

RNA extraction from frozen tissues was carried out following our established protocol [40]. Frozen callus (or equivalent femoral sections of sham mice) and quadriceps tissue samples were cryogenically ground using a Cryomill (Retsh GmbH, Germany). TRIzol reagent (Invitrogen, Thermo Fisher Scientific, Wilmington, MA) was added to an aliquot of tissue powder followed by homogenization using a Precellys Evolution Homogenizer and Cryolys Evolution (Bertin Technologies SAS, France). RNA extraction was carried out using TRIzol RNA extraction method combined with the miRNeasy Mini Kit total RNA purification procedures (QIAGEN Inc., Germantown, MD, USA). The quantity and quality of RNA was determined using the NanoDrop 2000 spectrophotometer (Thermo Fisher, Wilmington, DE, USA) and the Agilent Tapestation 2200 (Agilient Technologies, Inc., Santa Clara, CA, USA), respectively. The purified RNA samples were stored at -80°C for future use.

### miRNA-sequencing assay

The small RNA libraries were prepared as reported previously [41] using TruSeq Small RNA Sample Preparation Kit (Illumina, San Diego, CA, USA) with multiplexing adapters, following the user guide. Prepared libraries were pooled, size selected for a product of 145–160 bp, and gel purified, quantified and pooled for single-end sequencing on Illumina NextSeq 500 Sequencer.

### Tissue proteomics

The Olink assays on callus tissue samples were performed using Olink Target 96 Mouse Exploratory (v.3801) panel (Olink Proteomics, Watertown, MA). Protein concentrations on homogenized were checked using Qubit (ThermoFisher Scientific, Waltham, MA, USA) and equal quantities were loaded for measurements using Proximity Extension Assay technology (PEA) according to manufacturer’s instructions. Each sample was spiked with quality controls to monitor the incubation, extension, and detection steps of the assay. Additionally, samples representing external, negative, and inter-plate controls were included in each analysis run. From raw data, real-time PCR cycle threshold (Ct) values were extracted using the Fluidigm RT-PCR analysis software at a quality threshold of 0.5 and linear baseline correction. Ct values were further processed using the Olink NPX manager software (Olink). Here, log2-transformed Ct values from each sample and analyte were normalized based on spiked-in extension controls and scale-inverted to obtain Normalized log2 scaled Protein eXpression (NPX) values. NPX values were further adjusted based on the median of inter plate controls (IPC) for each protein and intensity median scaled between all samples and plates.

### Data analysis

The base calling log (BCL) files were converted to FASTQ files using BCL2FASTQ 1.8.4 from Illumina and data was subsequently demultiplexed. Bioinformatics analyses were performed using the CLC Genomics Workbench 20.0.4 software (QIAGEN). Initial quality check and trimming on FastQ files was performed using default settings. The total number of known miRNAs was annotated using miRbase- 22.1. Read counts of mature miRNA were then normalized using the trimmed mean of M values (TMM) method (S1 Table) that adjusts for the library size. Mature miRNA expression values were subjected to differential expression in two groups. Statistical comparisons were performed for each of the group with GRD-Sham group as a control for callus and quadriceps tissue, respectively. The miRNA (p<0.05) were used for core miRNA analysis in Ingenuity pathway analysis (IPA) (Qiagen) using default setting. For diseases and biofunction enrichment, all cancer or tumor related biofunctions were removed from the final tables. Predicted miRNA targets were determined using the Ingenuity Pathway Analysis (IPA, Qiagen, Hilden, Germany) miRNA Target Filter feature. IPA predicts miRNA-mRNA interactions at 3 confidence levels: experimentally observed, high confidence, and moderate confidence. Pairings from previously published mRNA data from quadriceps [31] and callus [32] were used and filtered for inverse expression. and predicted the miRNA-mRNA interactions using TargetScan, TarBase, miRecords, and the Ingenuity® Knowledge Base. ThemiRNA for signaling pathways for muscular and skeletal disorders were included in the final analysis. Graphpad Prism version 7 (Dotmatics, Boston, MA, USA) was used for plotting the graphs. Venny was used for depicting unique and shared sets of miRNA/gene lists and generating Venn diagrams [42].

## Results

Previous work from our lab examined samples from RR4 mission focusing on musculoskeletal components and all data outputs are summarized as S2A Table. Of particular importance for these studies are our micro-computed tomography bone healing findings detailed in Chakraborty et al. [32]. Briefly, at the time point examined in this mission, complete bridging of the bone defect was not observed in the FLT-Surgery or GRD-Surgery controls whereas bridging was observed in mice treated with bone morphogenetic protein 2 (BMP2) (these mice were not examined in the present study). However, when comparing saline treated FLT-Surgery to GRD-Surgery (the mice examined in the present study), FLT resulted in >50% increase in trabecular separation and a >50% reduction in trabecular connectivity although no differences in total callus volume or the percentage on mineralized callus were observed.

In the previous molecular analysis using gene-metabolite network, pro-apoptotic and anti-migration network was identified post-surgery as a result flight. The mouse muscle was associated with inhibited bioenergetics because of spaceflight. This complex regulation of gene expression and metabolism likely involve miRNA because of their ubiquitous role [43] and therefore our current study involves logical next steps of complementing with miRNA analysis associated with FLT and/or surgery. Here, we are implementing a comprehensive molecular systems biology approach, by combining gene-epigene analysis to help elucidate pathways affected by FLT conditions.

### miRNAs sequencing data

**Fig 2:**
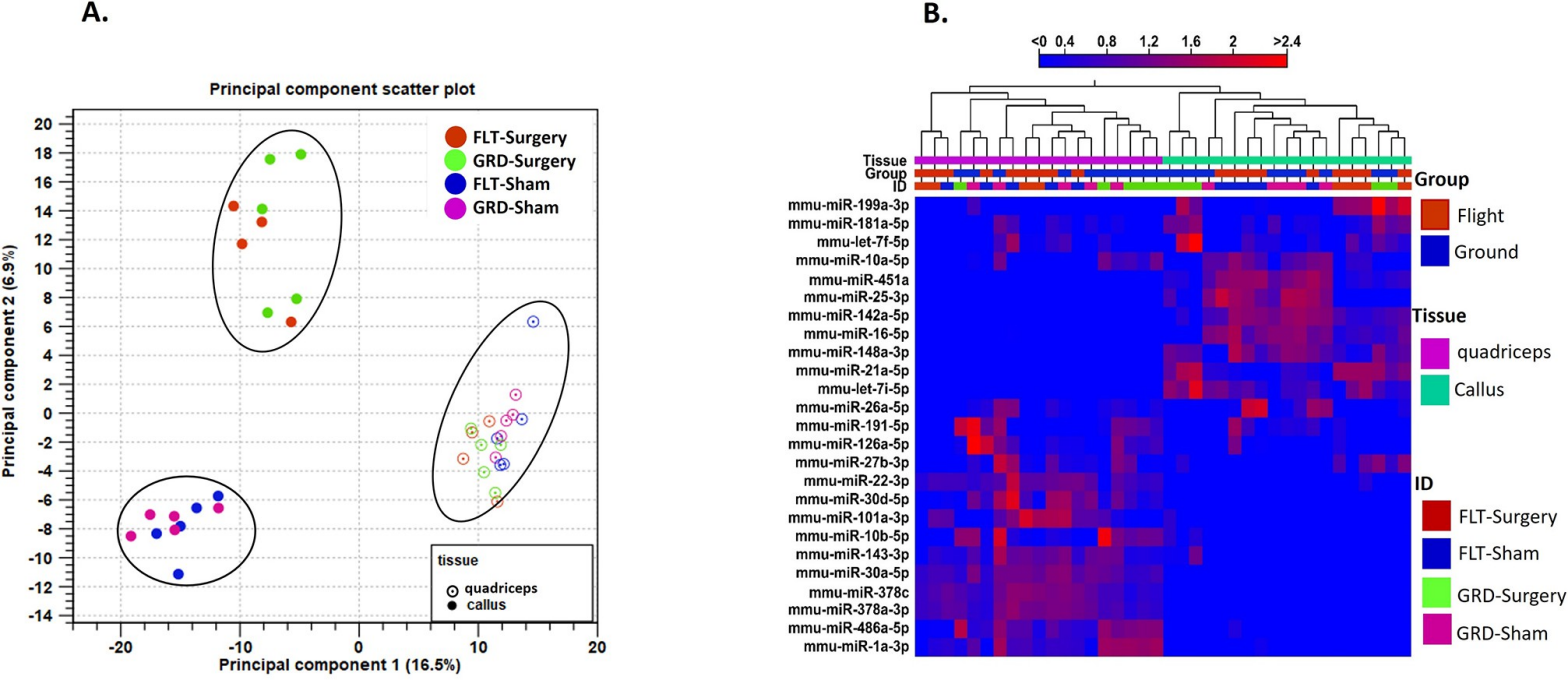
miRNA expression data. (a) Sample distribution within the first two principal components obtained from principal component analysis (PCA) along with the percentage of variance explained in each dimension; (b) Heatmap generated using hierarchical clustering based on the Top 25 features of miRNA expression pattern showing the relative differences within and among the biological replicates from callus and quadriceps tissue.

RNA integrity number (RIN) is normally not considered as a degradation indication for these small RNA [44] as these are small single stranded RNA. The average RIN value was 2.0 (SD  = 0.65) for bone tissue and 4.3 (SD = 1.4) for muscle tissue. The callus samples yielded higher number of sequencing reads as compared to quadriceps samples. (S1a Fig). However, different read depth did not lead to mapping differences in these tissues and all groups of mice (S1b Fig). On average, callus tissue samples mapped to 550 mature miRNAs and quadriceps tissue mapped to an average of 516 mature miRNAs. The first two components of principal component analysis (PCA) separated callus and quadriceps tissues based on the miRNA profile (Fig 2A) and (S2A Fig). There was an overall strong surgery effect as evidenced by separation of surgery (GRD-Surgery and FLT-Surgery) from the sham samples (GRD-Sham and FLT-Sham) in bone tissue.

**Fig 3:**
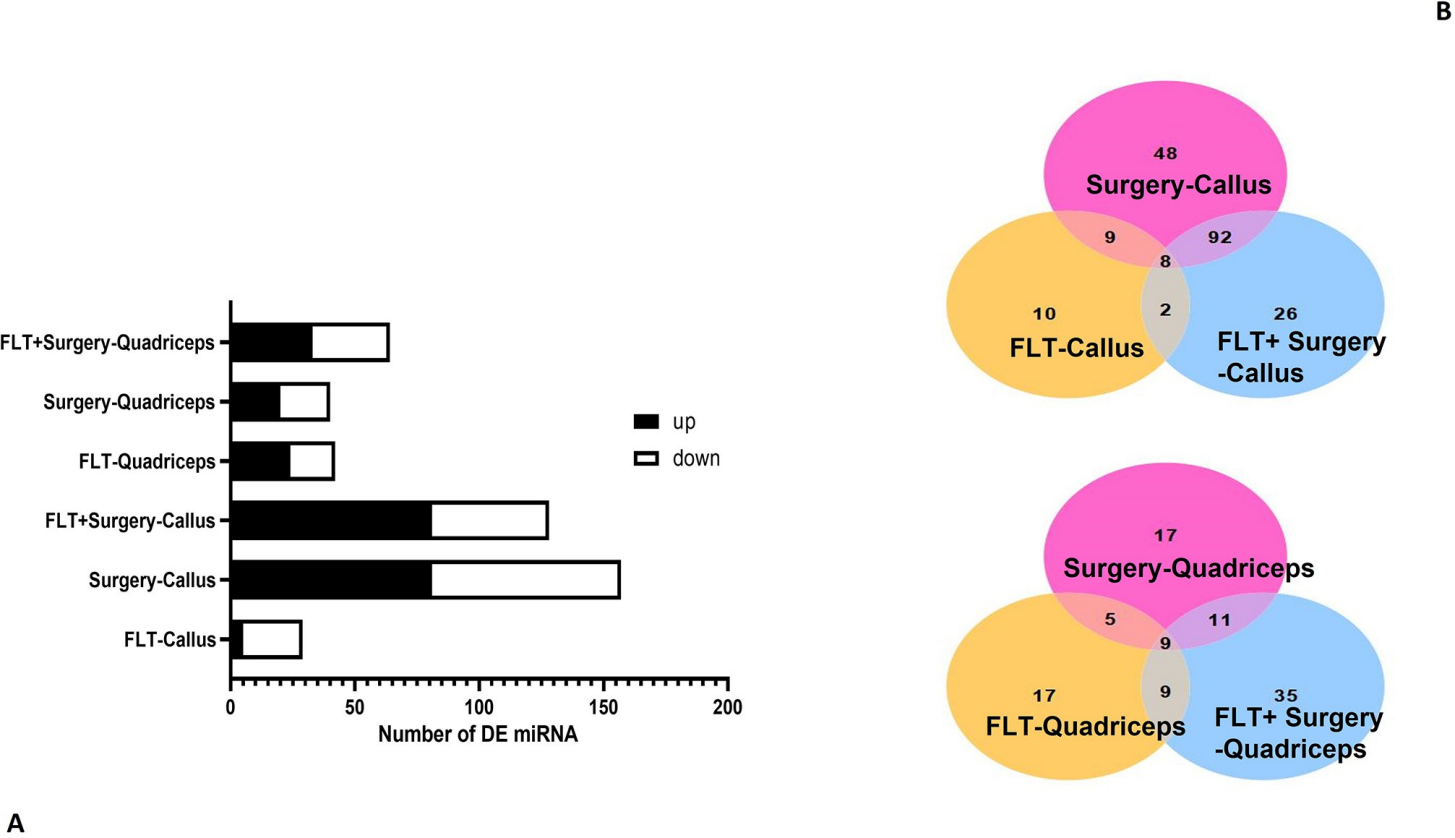
(a) Differentially expressed miRNA generated by comparing the groups (FLT-Sham, FLT-Surgery and GRD-Surgery) to the GRD-Sham group for the bone and quadriceps tissues. The microgravity effect is shown when FLT-Sham is compared to GRD-Sham; surgery effect is shown when GRD-Surgery is compared to GRD-Sham and surgery plus microgravity effects are reflected when FLT-Surgery is compared to GRD-Sham baseline samples. The current graph shows the number of miRNA at cut off p<0.05. (b) Venn Diagram representing the overlap between three differential analysis groups. The microgravity effect is shown when FLT-Sham is compared to GRD-Sham; surgery effect is shown when GRD-Surgery is compared to GRD-Sham and surgery plus microgravity effects are reflected when FLT-Surgery is compared to GRD-Sham samples for (i) bone tissue and (ii) quadriceps tissue. The overlap shows number of differentially expressed miRNA significant at cut off of p<0.05. (FLT: Spaceflight, GRD: Ground).

Surgery had highest number of differentially expressed miRNA (DEMi) (81 upregulated and 76 downregulated) in comparison to microgravity effect (5 upregulated and 24 downregulated). The combination of flight and surgery had 81 upregulated and 47 downregulated miRNA. There were 24 upregulated and 18 downregulated miRNA post-surgery, whereas 20 upregulated and 20 downregulated DEMi corresponded to microgravity effects in case of muscle tissue. The combination of FLT and surgery had 33 upregulated and 31 downregulated miRNA in quadriceps tissue (S2B Table and Fig 3A). There were 8 common miRNA in bone (*mmu-miR-451a*, *mmu-miR-592-5p*, *mmu-miR-712-5p*, *mmu-miR-33-5p*, *mmu-miR-365-3p*, *mmu-miR-6516-3p*,*mmu-miR-205-5p*) and 9 in quadriceps (*mmu-miR-208b-3p*, *mmu-miR-6240*, *mmu-miR-714*, *mmu-miR-712-5p*, *mmu-miR-2137*, *mmu-miR-10a-5p*, *mmu-miR-3470a*, *mmu-miR-146b-5p*, *mmu-miR-6238*) when FLT, surgery, and FLT plus surgery effects were studied (Fig 3B).

### miRNA network analysis

The miRNA expression data post-surgery showed that bone tissue was involved in 23 networks, whereas the quadriceps tissue had 11 associated networks. The FLT alone had 12 and 11 networks for bone and quadriceps, respectively, whereas FLT plus surgery had 12 and 19 for callus and quadriceps, respectively. Bone tissue was enriched in the biofunctions such as leukopoiesis, granulopoiesis, myelopoiesis of leukocytes, differentiation of myeloid leukocytes, differentiation of progenitor cells, differentiation of hematopoietic progenitor cells, metastasis of cells, neoplasia of cells, apoptosis, necrosis, and activation of migration of cells, cell viability, fibrosis and tubulation of cells (Table 1). All these biofunctions were either impacted by surgery or FLT plus surgery combination but were not observed in FLT only group. Similar biofunctions with somewhat-diluted effect was also observed for quadriceps tissue where leukopoiesis, differentiation of hematopoietic progenitor cells, apoptosis, necrosis, migration of cells and cell viability were enriched. All these biofunctions were limited to surgery and surgery plus FLT groups (Table 1). The clustering of miRNA involved in a few of these biofunctions such as leukopoiesis, apoptosis, and migration of cells for callus and quadriceps is shown as S2 Fig. S3 Table list all significant miRNA at p value 0.05. For the apoptosis biofunction, most of the miRNA were common for surgery and FLT plus surgery conditions in bone tissue, though the expression pattern trends were similar in callus and quadriceps samples. Similar phenomenon was observed for migration of cells and many miRNAs were found to be enriched in callus tissue with diluted effect in quadriceps sample groups.

**Table 1:** Diseases and biofunctions affected by differentially expressed miRNA (DEmi) in callus and quadriceps using miRNA analysis.

Diseases and Biological Functions	Surgery +Flight	Surgery	Flight	Surgery +Flight	Surgery	Flight
	Callus			Quad		
Leukopoiesis	-2.6	-2.6		-0.4		
Granulopoiesis	-2.4	-2.4				
Myelopoiesis of leukocytes	-2.4	-2.4				
Differentiation of myeloid leukocytes	-2.4	-2.4				
Differentiation of progenitor cells	-2.4					
Differentiation of hematopoietic progenitor cells	-2.2			-1.2		
Metastasis of cells	-1.1	-1.1				
Neoplasia of cells	-0.5	1.0				
Apoptosis	-0.4	-1.1		-0.4	-1.8	
Necrosis	-0.3	-1.0		-0.5	-1.5	
Migration of cells	0.6	1.1		1.0		
Cell viability	1.1	0.1		0.6		
Fibrosis		2.0				
Tubulation of cells		1.1				

IPA miRNA core analysis was conducted on DEmi from each of the respective group (FLT-Sham/GRD-Sham, GRD-Surgery/GRD-Sham, and FLT-Surgery/GRD-Sham). The data are represented as z-score that indicates a predicted activation or inhibition of a pathway. The miRNA in turn regulates multiple mRNA for pathways to be activated or inhibited. Values of |z|> 2 are considered significant. (FLT: Spaceflight, GRD: Ground). Blue and red bars are representing the Z score as represented by positive and negative values respectively.

### Microgravity effects using mRNA-miRNA integrative analysis

Experimentally validated targets perturbed because of microgravity were identified using the gene expression data previously generated from these groups. The expression of these targets was studied using the miRNA list from the sham group of bone and quadriceps tissue as well as surgery group from bone tissue (S3 Table). We identified 68 mRNA targets in bone and 87mRNA targets in quadriceps tissue as response to microgravity. There were 215 targeted mRNA identified in surgery bone tissue because of microgravity (S4 Table). Functional analysis of these lists indicated significant enrichment network categories including cellular functions such as cell cycle, cell death and survival, cellular development, cellular function and maintenance, cellular growth and proliferation, and cellular movement. Not only, lymphoid, and hematological system functions were affected but networks such as immune and inflammatory responses were affected as a response to microgravity.

**Fig 4:**
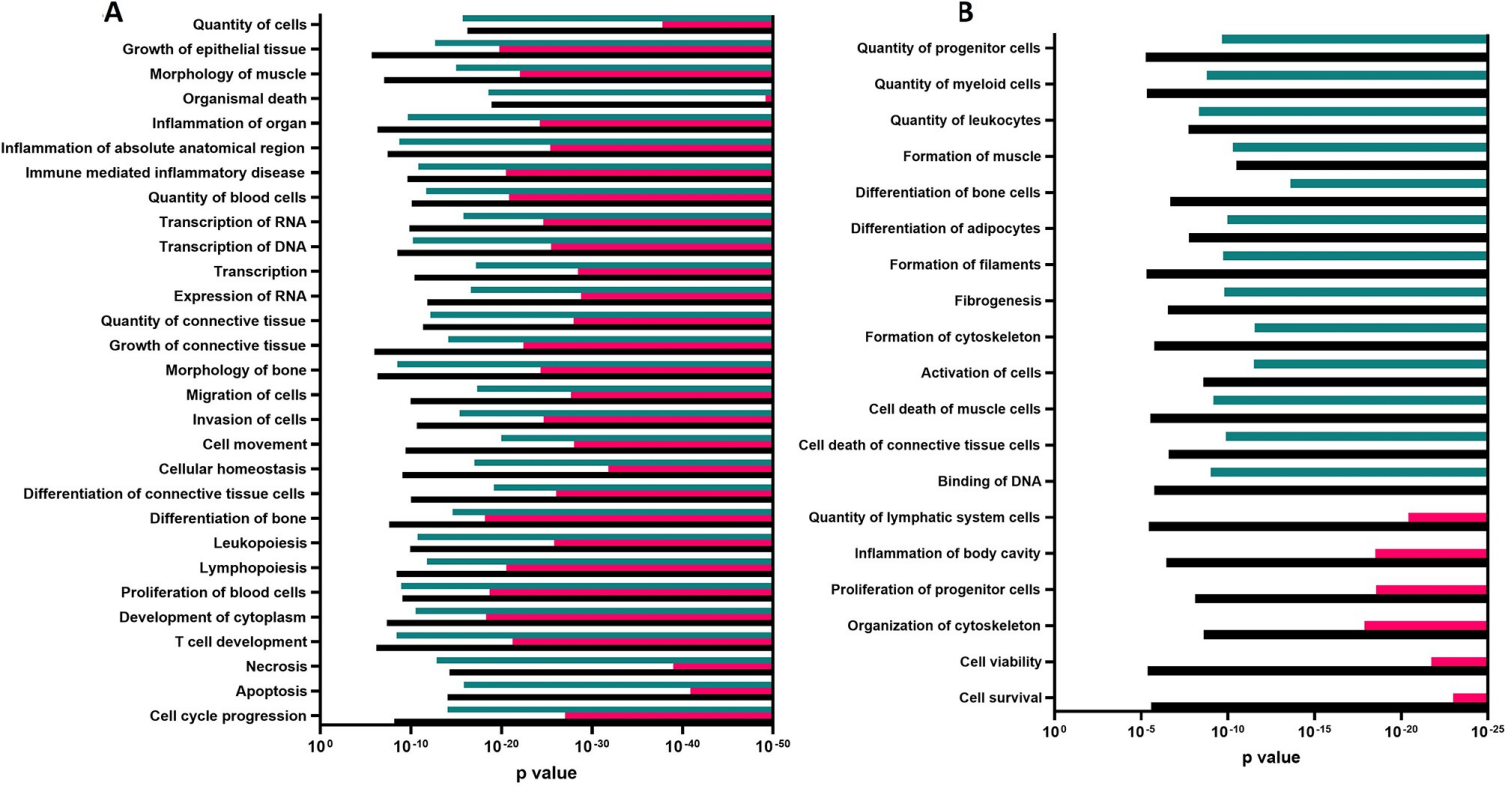
miRNA-mRNA target scan filter: Gene expression data published earlier (31, 32) was combined with miRNA data generated from current study. The integrative analysis gave a list of predicted biological functions that were affected because of microgravity. (a) Biological functions common in three groups: Microgravity effects that were common in all three groups i) sham bone, ii) sham quadriceps, and iii) surgery bone tissues are plotted with the p-values generated using the IPA analysis. (b) Biological functions common in ANY two groups Microgravity effects that were common in either of two groups i) sham bone; ii) sham quadriceps; and iii) surgery bone tissues are plotted with the p-values generated using the IPA analysis. Following groups are analyzed to study microgravity effects; callus-sham (Bone FLT-Sham to GRD Sham), quadriceps-sham (quadriceps FLT-Sham to GRD Sham), callus-surgery (Bone- FLT-Surgery to GRD Surgery).

**Fig 5:**
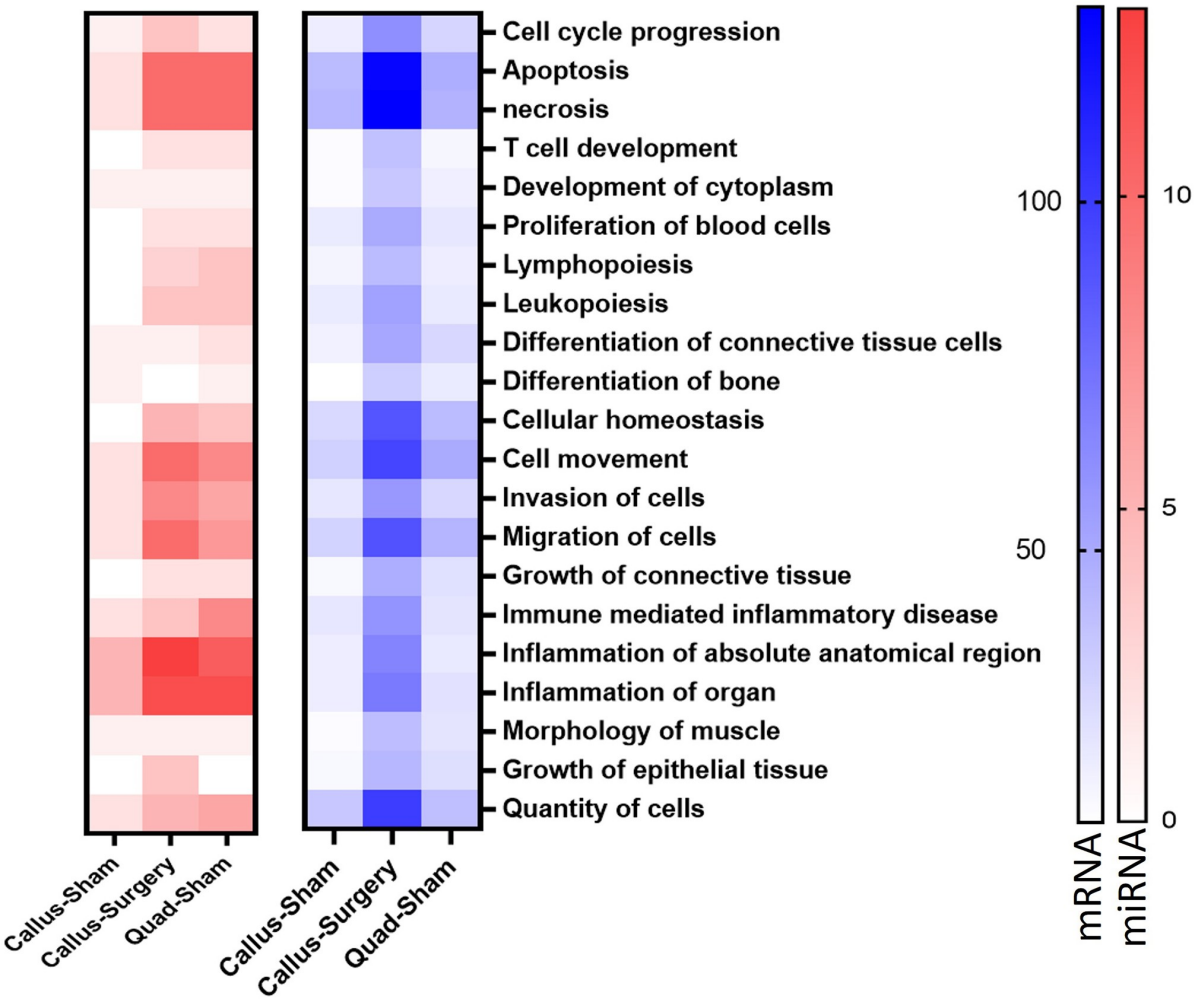
Heatmap representing number of miRNA and mRNA in biological networks affected by microgravity. The miRNA-mRNA integrated analysis predicted biological functions affected because of microgravity. The number of miRNA is shown as red panel heatmap and number of mRNA is shown as blue panel heatmap. Following groups are analyzed to study microgravity effects; callus-sham (bone FLT-Sham to GRD Sham), quadriceps-sham (quadriceps FLT-Sham to GRD Sham), callus-surgery (bone- FLT-Surgery to GRD Surgery).

*In silico* analysis of miRNA-mRNA target prediction confirmed that microgravity impacted surgery bone the most with lowest predicted p-values as compared to the bone or quadriceps sham groups (Fig 4A). This observation was strengthened by observed number of mRNA and miRNA in each of this tissue. We parsed out the number of mRNA and miRNA separately for the shortlisted biofunctions that are affected by microgravity conditions and callus-surgery samples had the largest number of miRNAs as well mRNA, followed by the quad-sham samples. Combining the data showed that microgravity had larger effect on for quad-sham as compared to callus-sham (Fig 5A). The microgravity led to expression changes in unique mRNA/miRNA in each of the conditions as represented by the Venn diagram for necrosis biological function (S3 Fig). The top functional annotations with maximum number of molecules were related to necrosis, apoptosis, cellular movement, quantity of cells, migration of cells and cellular homeostasis with 138, 135, 106, 105, 100, and 94 molecules, respectively (S5 Table). The maximum number of miRNAs belong to the functional annotation inflammation of absolute anatomical region with 13 miRNAs involved in bone-surgery group (S6 Table).

By an integrated approach of miRNA and mRNA, microgravity led biological network changes common in only bone tissue with and without surgery were identified. In addition, microgravity led biological network changes common only in sham bone and quadriceps were also reported. The predicted p- values for these functions were lowest for the callus-surgery group, followed by quad-sham and callus-sham groups (Fig 4B).

### Microgravity effects using protein analysis

**Fig 6:**
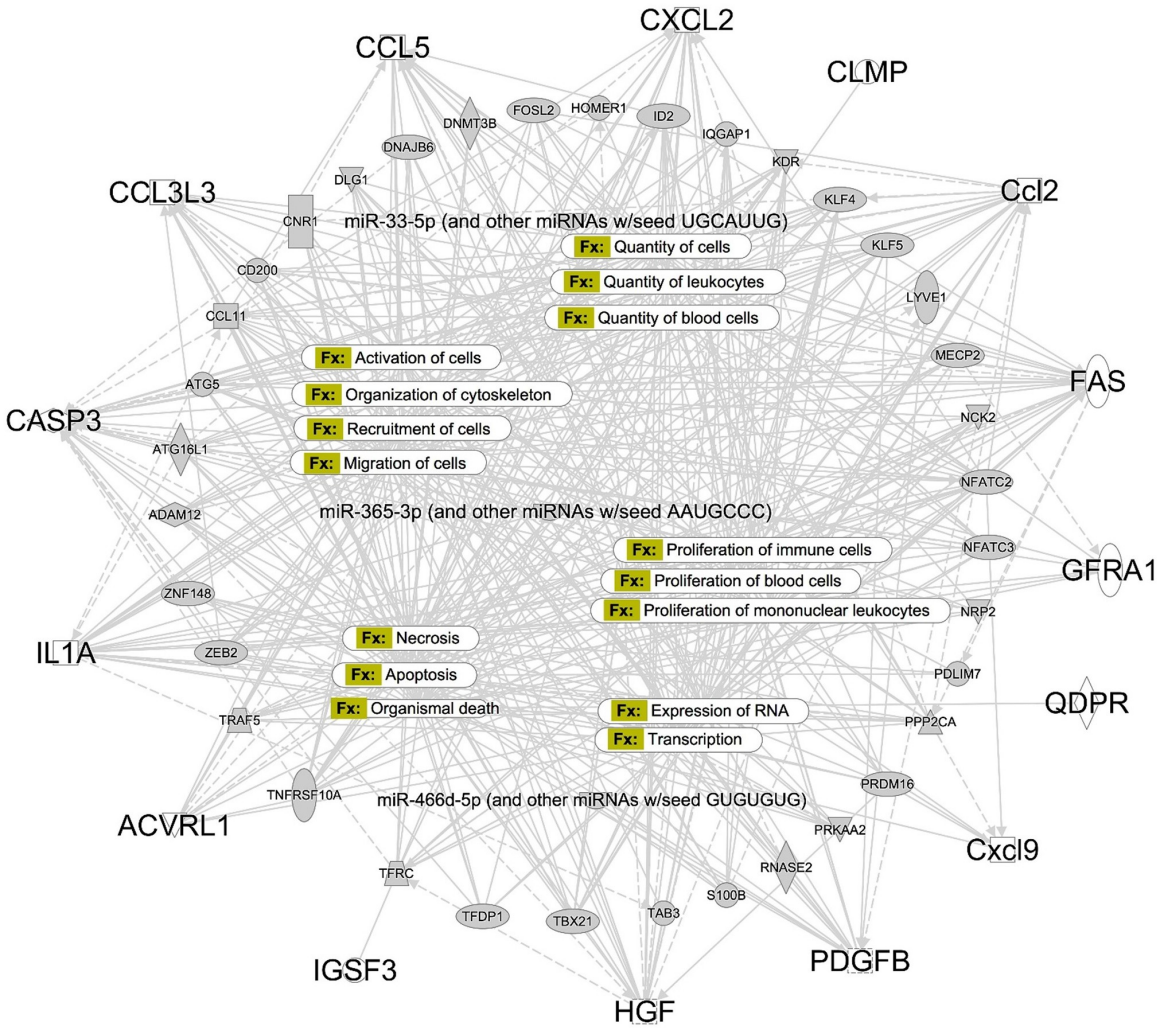
Microgravity effects using integrative protein, mRNA, and miRNA analysis. Top 15 diseases and function altered in bone tissue as a result of microgravity. The outer circle in the figure denotes the significantly different proteins from S3 Fig. These proteins were integrated with miRNA profile filter of 10 miRNA targeting 68 mRNA (center loop) targets. All the biological functions were listed in the middle showcasing functions such as cell death, cell number, cell migration, immune response, and gene expression changes. This figure is generated by IPA and Fx denotes disease and function, node shape for the genes is default settings from IPA.

The comparison of sham bone data from GRD and FLT showed a strong perturbation of proinflammatory cytokines and chemokines (S4 Fig). Integration of proteins data validated the findings from the gene-epigene network analyses. The main biological function with 40 molecules is organismal death followed by migration of cells with 37 molecules. The top 15 biological networks were linked to cell growth and proliferation, cellular assembly and organization, cell death, cell cycle control and cell signaling, control of gene expression, and immune and inflammatory response that were affected by the microgravity condition (Fig 6).

## Discussion

The bone healing process has been extensively studied using animal models [45, 46]. However, the complexity increases when this process is orchestrated in microgravity due to unloading conditions. Using a SBD mouse model, we have generated a set of differentially expressed miRNAs data in microgravity and ground conditions. We have integrated it to overlapping RNA data in respective group analysis to study intricate regulatory circuits. Recently, synergistic effects of miRNA, long noncoding RNA, RNA expression coupled with cellular responses have been observed with a combination of simulated microgravity and irradiation [47]. Notably, microRNAs are altered in the blood of astronauts who have spent time on the ISS [48]. This is likely due to the weightlessness of microgravity, which induces bone loss and muscle atrophy in astronauts, partly through the suppression of bone formation and the stimulation of bone resorption [49]. The impact of microgravity on the musculoskeletal system has been extensively reviewed in the literature, using rodent and primate models [50]. However, the mechanisms underlying muscle atrophy, which involves higher protein degradation compared to protein synthesis, warrant further investigation [51]. In the case of bone healing, the muscles not only supply oxygen and nutrients but are also capable of enriching the secretome post-injury, potentially holding therapeutic potential [52]. Here, our study is focused on studying gene-epigene in bone and muscle tissue as a unit of the musculoskeletal system [53].

This study focuses on studying the gene-epigene interactions in bone and muscle tissue as a unit of the musculoskeletal system, to fully understand the complex transcriptional networks involved. We have not only focused on miRNAs as key post-transcriptional regulators but also used miRNA-target gene prediction to comprehensively dissect the molecular mechanisms. This approach has provided a more complete picture, as expression changes in miRNA-only data may not be sufficient to understand the full complexity of the system. Astronauts experience a 20% reduction in skeletal muscle mass and loss of strength after just one month of microgravity exposure [13]. Our miRNA-mRNA integrative data indicates that cell death in muscle formation along with many other biological functions impacting stem cells were affected. Furthermore, depending on the stimulus and the cell’s developmental programming, muscle fibers and stem cells can undergo necrosis or apoptosis [54]. In surgical bone tissue, miRNA had the greatest impact, which was further supported by the mRNA target scan feature. A few mRNA were shown to be involved in co-regulation were *miR214-5p* targeting 18 mRNA, *miR700-5p* targeting 17 mRNA, *miR205-5p* targeting 12 mRNA, *miR425-3p* targeting 13 mRNA and *miR466-5p* targeting 36 mRNA, *miR34a-5p* targeting 70 mRNA and *miR16-5p* targeting 6 mRNA. Many of these miRNA have been studied in the fracture healing process, with involvement in steps like hypoxia, angiogenesis, bone resorption, osteoclastogenesis, mineralization, and osteogenesis [6]. These may be directly or indirectly involved in the regulation.

Fracture healing is impacted by osteoblast differentiation and osteogenesis processes [55]. Exposure to microgravity weakened the highly regulated differentiation procedure of osteocytes and osteoclasts, thus affecting the bone regeneration process. Our data corroborates with previous finding that microgravity delays stem cells growth and differentiation [13, 56, 57] potentially due to the altered energy demand during the austere condition since glycolysis pathways are known to be involved in microgravity conditions [58]. We identified previously reported miRNA such as miR208a-3p suppressing osteoblast differentiation [59], miR-1-3p involved in differentiation of stem cells [60]. MicroRNAs *miR-17*, *miR-23a*, *and miR-31* inhibits bone morphogenetic protein-2 (BMP2) mediated osteogenesis [56] and *in-silico* analysis of current data showed that these may be playing role in leukocyte generation and cell migration.

A recent study investigating hindlimb unloading as a musculoskeletal disuse model on ground showed an early surge of apoptosis and inflammation in the femoral osteocyte cells which reached a plateau at 5 to 15 days post unloading [61]. In simulated microgravity conditions, *miR-132-3p* has been reported to inhibit osteoblast differentiation and participate in bone loss regulation [62]. Our data showed the mouse equivalent of this miRNA with a putative role in apoptosis for both microgravity and surgery conditions in bone and quadriceps tissue. Similarly, quadriceps tissue showed significantly changed mouse equivalent of *miR-103* post-surgery that is known to inhibit osteoblast proliferation in simulated microgravity [63]. To study the microgravity effects on quadriceps tissue, miRNA-mRNA integrative analysis showed *let-7a-5p* targeting 18 mRNA, followed by *miR-17-5p* with 15, *miR298-5p* with 12, and *miR148a-3p* with 12 mRNA targets. *miR-17-5p* targets mRNA CDKN1A (cyclin dependent kinase inhibitor) and are known to have role in cell-cycle/ apoptosis [64].

Microgravity also results in altered immune response and several chemokines important for myeloid cell trafficking [65] (e.g., CXCL1, a potent neutrophil-recruiting chemokine) and the differentiation and multiplication of leukocytes (e.g., CXCL9) were significantly changed in FLT bone samples. Alterations in chemokines, including CCL2, CCL3, and CCL5, point to the involvement of chemoattractants in the migration and multiplication of inflammatory cells. Numerous of these processes, including adhesion and migration, proliferation, genetic expression, cytokine secretion, the generation of reactive oxygen species, and polarization, have been demonstrated to be critically dependent on inflammatory cells like macrophages and monocytes [66]. Further, bone protein data validated the findings that microgravity alters *miR200b-3p that* targets 16 mRNA including *wif1* that is an WNT inhibitory factor regulating WNT with its role in organismal death, quantity of cells, and apoptosis [67] The role of NF-κB in skeletal muscle atrophy and bone loss have been widely reported [68] and a wide array of genes are known to be associated with NF-κB signaling [69], we identified miRNA such as *miR-486-5p* and *miR-21-5p* that are known to regulate hematopoiesis via NF-κB signaling.

Skeletal muscle and bones Crosstalk, which is crucial for preserving homeostasis, is the integration of molecular signals that permits communication between muscle and bone. Our findings indicate a spatial and metabolic relationship between skeletal muscle and bone, and the combination of miRNA and mRNA expression profiles has made it possible to apply sophisticated prediction algorithms to examine the intricate relationships. This study does come with some shortcomings and need to be considered. The study was conducted on 9-week-old mice and is comparable to average age of astronauts in space and complete picture can only be studied with varying age groups of mice. It is important to point out that tissue sample examined as the fracture callus was the fracture callus plus bone and bone marrow and as the sham controls were comprised only of bone and bone marrow, the difference observed in ground or flight specimens should appropriately reflect this issue. Microgravity experiments are usually multi-group collaborative studies with accessibility issues mainly related to space constraints and high expense. In our work, the combination of microgravity and the SBD surgery model caused a number of molecular alterations in the musculoskeletal system; interestingly, many of these modifications were linked to comparable biological roles, albeit with different mRNA or miRNA drivers.

## Supporting information

S1 Fig (a) Read counts for miRNA: The sequencing files were demultiplexed using standard Illumina recommended pipeline. Error bars are introduced by showing scatter among side by side for all replicates. (b) Number of mapped mature miRNA (GRD: Ground, FLT: spaceflight).
(TIF)

S2 Fig A. (a) **Principal components estimate**: Results from PCA showing estimates of variance in the expression data where each bar corresponds to one factor. B. **Clustering of miRNA in different biofunctions:** A) Leukopoeisis; B) Apoptosis; and C) Migration of cells. Data showing three analysis groups (FLT-Sham/GRD-Sham, GRD-Surgery/GRD-Sham and FLT-Surgery/GRD-Sham for each of the (i) callus and (ii) quadriceps tissue. The blue and orange legend refers to predicted activation or inhibition as marked in Table 1.
(ZIP)

S3 Fig Venn diagram A representative Venn showing number of mRNAs and miRNAs involved in necrosis biofunction showcasing each group with unique mRNA/miRNA set towards a common biological function. The groups referred in the figure points to the microgravity effect on callus-sham quadriceps-sham, and callus-surgery tissue.
(TIF)

S4 Fig Microgravity effects on proteins in sham callus tissue: FDR corrected and statistically significant altered proteins in callus tissue when FLT-Sham is compared with GRD-Sham samples. These proteins were identified after Olink assays and y-axis is the Ct Values as calculated using NPX software. These proteins are identified using unpaired t-test with FDR correction using two-stage step-up (Benjamini, Krieger, and Yekutieli) method. (FLT: Spaceflight, GRD: Ground). The error bars most often.
(TIF)

S1 Table TMM normalized data for callus and quadriceps tissue. The tissues were normalized separately using CLC Genomics Workbench using CPM normalization function. (FLT: flight, GRD: Ground). Table contains data from each mouse labeled as W1 to W10; C denotes callus and Q denotes quadriceps).
(XLS)

S2 Table **A**: Published Musculoskeletal studies from Rodent Research Mission-4. The musculoskeletal components from the same mice have been studied using other assays and description citing those references are listed here. **B:** List of Differentially expressed miRNA (p value <0.05) from each of the group comparisons. Max group means is the maximum of the average transcript per million; log2 fold change: the logarithmic fold change.
(ZIP)

S3 Table Hierarchical clustering output file based on p value for 1) Leukopoiesis 2) Apoptosis 3) migration of cells biofunctions respectively. The data is reported as p value and pink highlighted are significant at p<0.05.
(XLSX)

S4 Table Three lists showing number of mRNA targets altered by miRNA using Ingenuity pathway analysis. Following groups are analyzed to study microgravity effects; callus-sham, callus-surgery and quadriceps-sham. The settings of miRNA profile filter are discussed in method section of the manuscript. List 1 (callus-sham) callus_FLT_Sham/GRD_Sham; List 2 (callus-surgery) callus_FLT Surgery_GRD Surgery; and List3 (quadriceps-sham) Quad_FLT Sham_GRD Sham. (FLT: Spaceflight, GRD: Ground).
(XLSX)

S5 Table miRNA target filter generated list for each of the following group affected by microgravity; 1) callus-sham (FLT_Sham/GRD_Sham); 2) callus-surgery (FLT Surgery_GRD surgery); and 3) quadriceps-sham (FLT Sham_GRD sham). (FLT: Spaceflight, GRD: Ground. Expr: expression).
(XLSX)

S6 Table Functional annotation and molecules involved in miRNA-mRNA integrative analysis. Here microgravity effects are studies in callus sham group, callus surgery group and quadriceps sham group as analyzed using IPA.
(XLSX)
