# Supplementary figures and images for: Microgravity’s effects on miRNA-mRNA regulatory networks in a mouse model of segmental bone defects
Source: PLoS One. 2024 Dec 2;19(12):e0313768. doi: 10.1371/journal.pone.0313768 (PMC11611151; doi:10.1371/journal.pone.0313768)

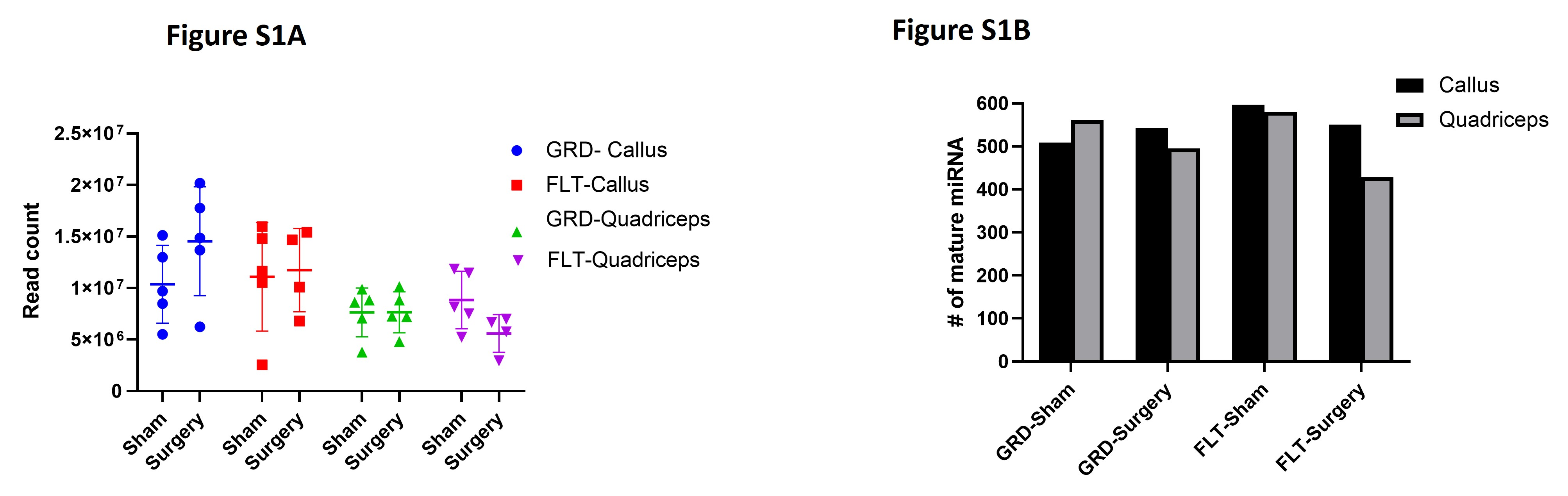

Supplement: S1 Fig — (a) Read counts for miRNA: The sequencing files were demultiplexed using standard Illumina recommended pipeline. Error bars are introduced by showing scatter among side by side for all replicates. (b) Number of mapped mature miRNA (GRD: Ground, FLT: spaceflight). (TIF) [file pone.0313768.s001.tif]

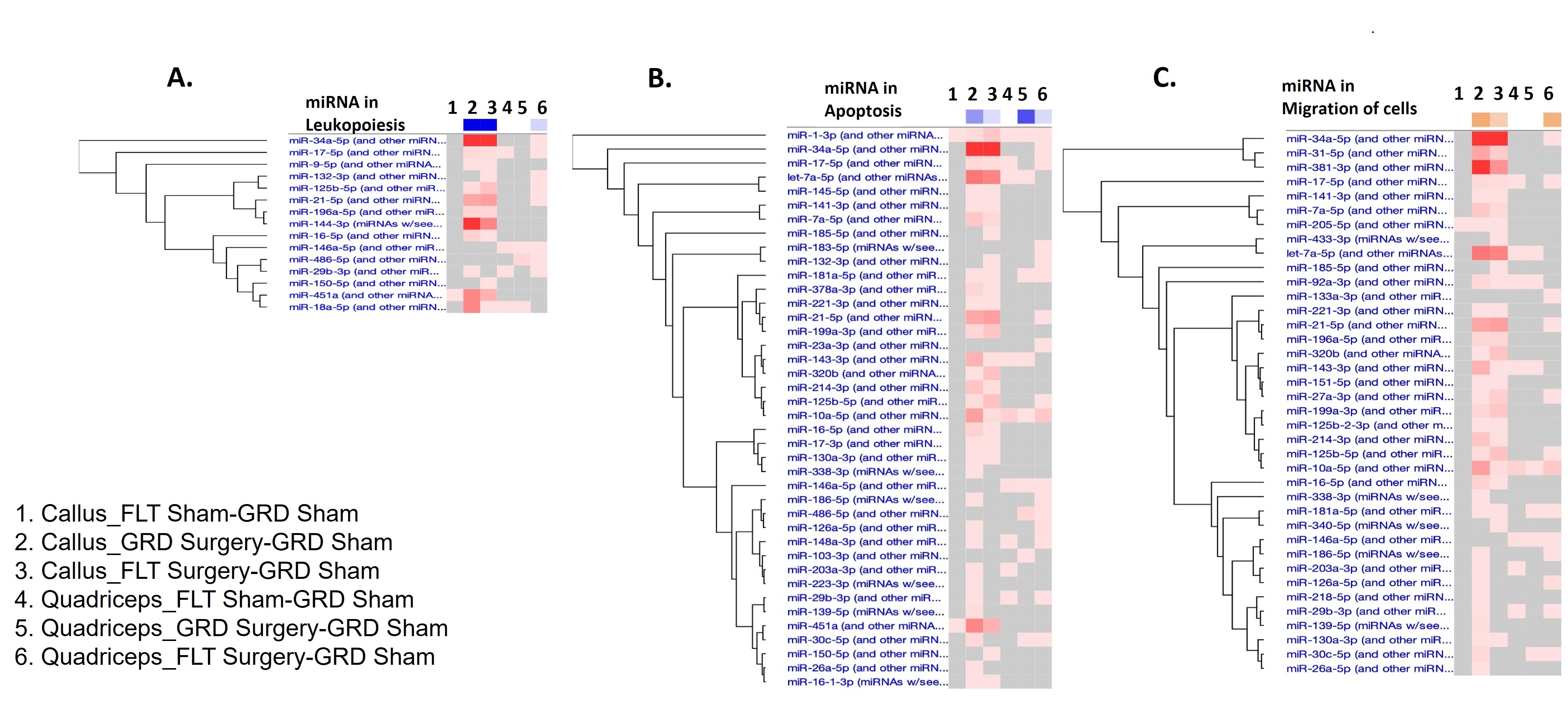

Supplement: S2 Fig — A. (a) Principal components estimate: Results from PCA showing estimates of variance in the expression data where each bar corresponds to one factor. B. Clustering of miRNA in different biofunctions: A) Leukopoeisis; B) Apoptosis; and C) Migration of cells. Data showing three analysis groups (FLT-Sham/GRD-Sham, GRD-Surgery/GRD-Sham and FLT-Surgery/GRD-Sham for each of the (i) callus and (ii) quadriceps tissue. The blue and orange legend refers to predicted activation or inhibition as marked in Table 1. (ZIP) [file pone.0313768.s002.zip › Figure S2B.tif]

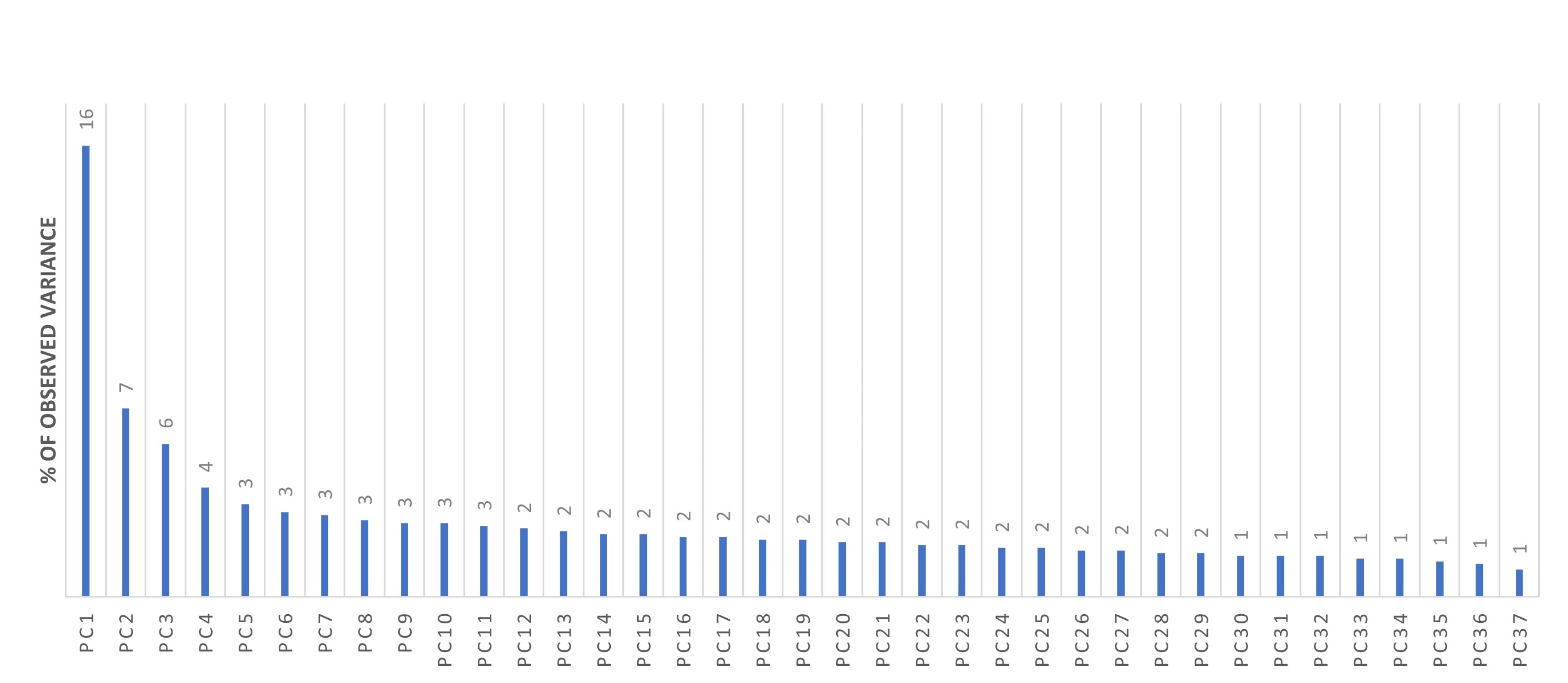

Supplement: S2 Fig — A. (a) Principal components estimate: Results from PCA showing estimates of variance in the expression data where each bar corresponds to one factor. B. Clustering of miRNA in different biofunctions: A) Leukopoeisis; B) Apoptosis; and C) Migration of cells. Data showing three analysis groups (FLT-Sham/GRD-Sham, GRD-Surgery/GRD-Sham and FLT-Surgery/GRD-Sham for each of the (i) callus and (ii) quadriceps tissue. The blue and orange legend refers to predicted activation or inhibition as marked in Table 1. (ZIP) [file pone.0313768.s002.zip › Figure S2A.tif]

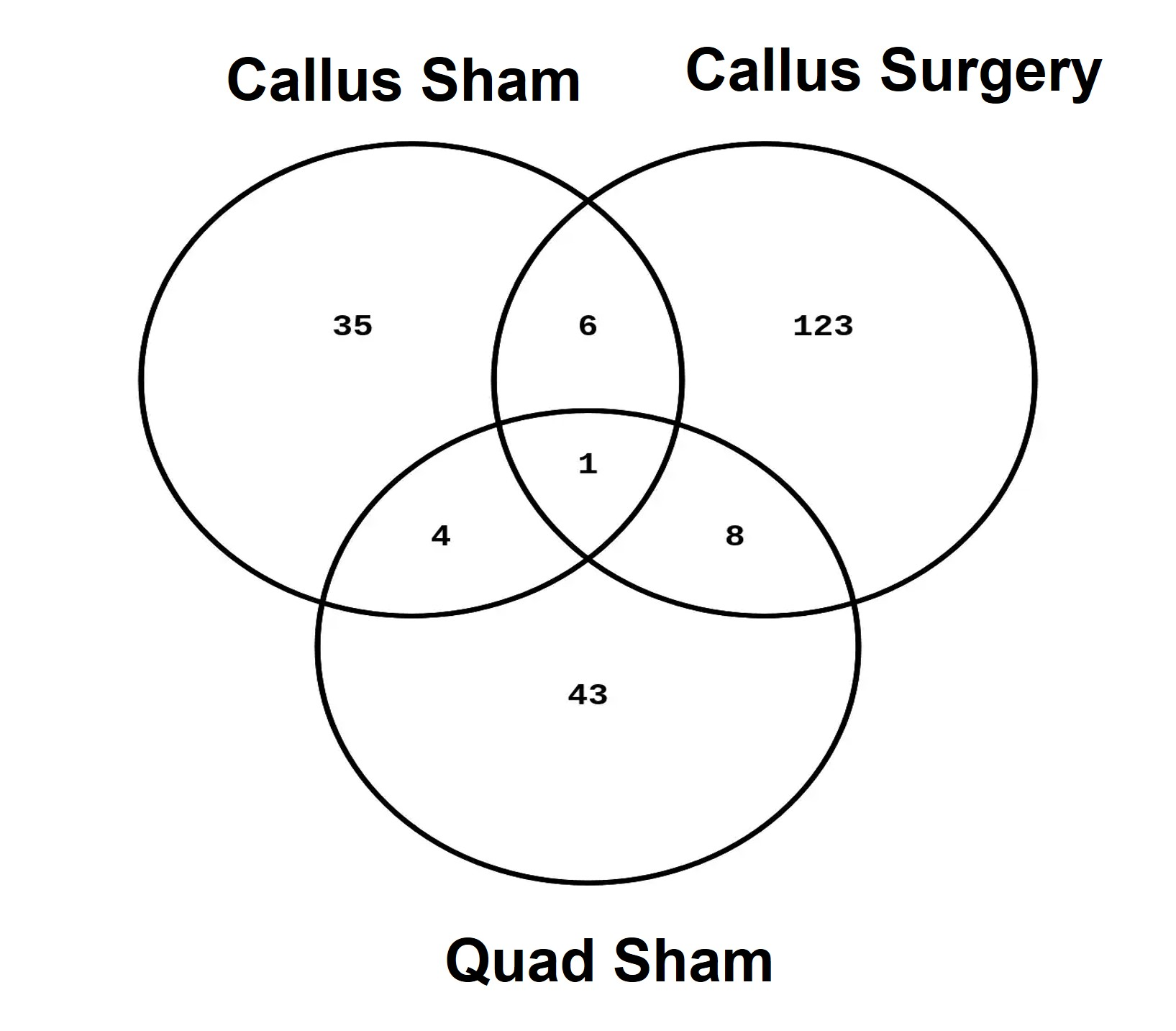

Supplement: S3 Fig — The groups referred in the figure points to the microgravity effect on callus-sham quadriceps-sham, and callus-surgery tissue. (TIF) [file pone.0313768.s003.tif]

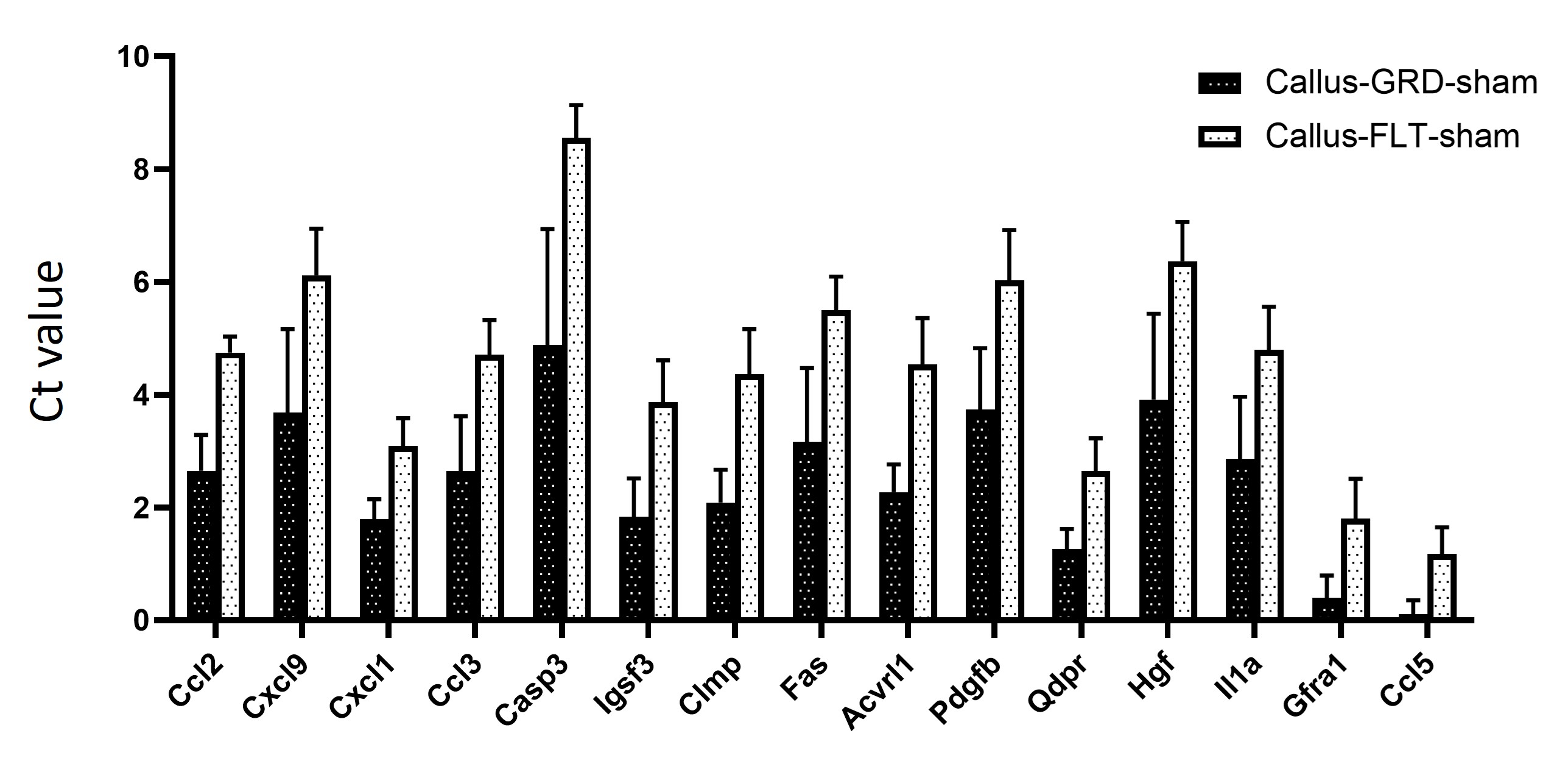

Supplement: S4 Fig — These proteins were identified after Olink assays and y-axis is the Ct Values as calculated using NPX software. These proteins are identified using unpaired t-test with FDR correction using two-stage step-up (Benjamini, Krieger, and Yekutieli) method. (FLT: Spaceflight, GRD: Ground). The error bars most often. (TIF) [file pone.0313768.s004.tif]
